# Association between antenatal depression and low birthweight in a developing country

**DOI:** 10.1111/j.1600-0447.2006.00950.x

**Published:** 2007-06-01

**Authors:** A Rahman, J Bunn, H Lovel, F Creed

**Affiliations:** 1 Department of Psychological Medicine, University of Manchester Manchester, UK; 2 Human Development Research Foundation Islamabad, Pakistan; 3 Department of Tropical Child Health, Liverpool School of Tropical Child Health Liverpool; 4 WHO Collaborating Centre for Primary Care Manchester, UK; 5 Department of Psychological Medicine, University of Manchester Manchester, UK

**Keywords:** mental health, postpartum depression, malnutrition, child development, developing countries

## Abstract

Rahman A, Bunn J, Lovel H, Creed F. Association between antenatal depression and low birthweight in a developing country.

**Objective:**

There is a high prevalence of depression in south Asian women. We aimed to examine the association between antenatal depression and low birthweight (LBW) in infants in a rural community in Rawalpindi, Pakistan.

**Method:**

A total of 143 physically healthy mothers with ICD-10 depression in the third trimester of pregnancy and 147 non-depressed mothers of similar gestation were followed from birth. Infant weight was measured and information collected on socioeconomic status, maternal body-mass index and sociodemographic factors.

**Results:**

Infants of depressed mothers had lower birthweight (mean 2910 g) than infants of non-depressed mothers (mean 3022 g). The relative risk for LBW (≤2500 g) in infants of depressed mothers was 1.9 (95% CI 1.3–2.9). The association remained significant after adjustment for confounders by multivariate analyses.

**Conclusion:**

Low birthweight is a major public health problem in developing countries. Maternal depression during pregnancy predicts LBW. Interventions aimed at maternal depression may help improve infant outcomes.

## Significant outcome

- Maternal depression during pregnancy, a treatable disorder, is independently associated with low birthweight.

## Limitations

- Small sample size from only one subdistrict means results should be generalized with caution.
- Data was not collected on maternal anaemia as a potential confounder.

## Introduction

Low birthweight (LBW) (weight at birth of less than 2500 g) is associated with greatly increased risk of mortality and morbidity in infants (1). It is also a risk factor for emotional problems (2) and psychotic illness (3) in later life. Nearly a third of all infants in south Asia are born with LBW – the determinants of this high incidence are not fully understood (4). In developed countries, research has shown that maternal psychosocial stress and anxiety during pregnancy are associated with poor birth outcomes including LBW, prematurity and miscarriage (5–8). This has led to growing interests in the effects of stress-related psychoneuroendocrine processes in human pregnancy on foetal developmental and health outcomes (9, 10). However, the area remains under-researched in developing countries, where depressive disorder during the third trimester of pregnancy occurs in up to 25% of mothers in South Asia (11), and the level of LBW is more than four times the level (7%) in industrialized countries (1).

We have previously shown a strong association between maternal depression and later growth retardation in infants in Pakistan (12). While we found an association of antenatal depression with LBW, we did not examine if the association was independent of the effects of other antenatal risk factors such as maternal age, family size, socioeconomic and nutritional status. A recent study from India has found that self-reported maternal psychological morbidity is independently associated with LBW (13). However, this study relies on a self-report screening questionnaire to measure mental distress. No study from the developing world has examined the effects of operationally defined depression during pregnancy on infant outcomes.

### Aims of the study

In this paper we aim to test the hypothesis that, in the setting of a low-income Pakistani community ICD-10 defined maternal depressive disorder in the third trimester of pregnancy is associated with LBW after controlling for possible confounders.

## Material and methods

### Study population

The study was performed in a rural subdistrict of Rawalpindi, Pakistan. The study area comprised 10 Union Councils (each consisting of 5–10 villages) of the subdistrict, which covered about 500 km^2^ and included a population of 150 000. The area is predominantly agrarian, food-sufficient and not endemic for malaria or iodine-deficiency. The sample consisted of all women aged 17–40 years in their third trimester of pregnancy, recruited from the study area over a 4-month period. Subjects were identified by obtaining official lists from 120 government-employed Lady Health Workers (LHWs) working in the area, who routinely collect data on new pregnancies including an estimate of the gestational age (based on the last menstrual period). Each LHW covers 1000 population (approximately 130 households), visiting about 30–35 houses per week. As an additional incentive, they were paid a small amount for every subject identified. In some villages these data were verified by employing local traditional birth attendants and key informants to carry out door-to-door surveys. Thus, near-full coverage of the study area was achieved. All identified women were married, and were included in the study if they did not have a physical illness for which they were under treatment, and had had an uneventful pregnancy. Women with severe depression or other mental disorder were excluded. Stillbirths, infants who died before reaching their first birthday or born with a congenital abnormality were excluded from the study, as were mothers who gave birth prematurely ( < 37 weeks according to gestational age calculated by LHWs). None of the mothers in the sample smoked. None were receiving antidepressant medication.

### Procedures

#### Assessment of maternal depression and disability

Informed written consent was obtained from all participants after explaining the study and providing a detailed information sheet (local health workers read out and explained the information to non-literate participants and obtained written consent on their behalf). Mental state was assessed in the third pregnancy trimester using the Schedules for Clinical Assessment in Neuropsychiatry (SCAN) (14), developed by the World Health Organization as an internationally validated semi-structured interview generating ICD-10 diagnoses of Depressive Disorder (15). All interviews were carried out by two trained and experienced clinicians and inter-rater reliability was established between them by independent assessment of 20 women (Kappa = 0.90).

#### Assessment of birthweight

Birthweight was measured to the nearest 0.1 kg within 2 days of birth by the area's Lady Health Worker using a portable 25 kg spring balance Salter Scale (Salter England, West Bromwich, UK). The scale was similar to that used routinely and the LHWs are carefully trained in its use. All measurements were made using new spring balance weighing scales with bowl bags standardized with a 10 kg weight. The standard cut-off for LBW in infants is 2500 g or less (16).

#### Assessment of economic and social status

We compared depressed and non-depressed mothers in terms of ownership of household assets using the World Bank Assets Questionnaire for Pakistan (17). We also enquired if the household was in debt, had enough money to buy food and basic household needs. The LHWs, who lived in the same locality and had intimate knowledge about the families being studied, rated the household on a five-point Likert scale ranging from 1 (richest) to 5 (poorest). A single dichotomous variable of ‘relative’ poverty was created by combining all of these measures. For example, subjects who were both in debt and rated below 3 on the LHWs’ five-point Likert scale were classified as being ‘relatively poor’.

We asked each mother if she was given a lump-sum amount of money for day-to-day household expenses, and whether she could take independent decisions about its use. Mothers who answered ‘yes’ to both questions were classified as financially empowered within the household.

#### Assessment of other risk factors

We calculated data on the following potential confounders, which were dichotomized on an a-priori basis as: (1) Maternal age – younger maternal age, ≤20 years vs. 21 years or older; older maternal age, ≥30 years vs. 29 years or younger. (2) Low maternal body-mass index (BMI), at 8 weeks postnatal, defined as ≤18.5, vs. BMI above 18.5. (3) Less than 4 years vs. at least 4 years of primary education for each parent, as many Pakistani female children from low-income families stop attending primary school after 4 years. (4) Two or more children under 7 years vs. < 2 children under age 7. (5) Four or more children, vs. less than 4 children. This cut-off was chosen because we believed that low-income families with ≥4 children might have additional difficulties such as financial constraints and overcrowding. (6) Nuclear family (parents and children only) or extended family (three generations, or one or both parents with married sons, their wives and children). (7) At least one antenatal consultation with health personnel vs. no contact with any health personnel during pregnancy.

### Statistical analysis

We first compared depressed and non-depressed groups using chi-squared and Mann–Whitney *U*-tests. Mean differences in birthweight between exposed (infants of antenatally depressed mothers) and non-exposed group (infants of antenatally non-depressed mothers) were analysed. Univariate analyses were then carried out to estimate relative risks with 95% CIs of having LBW (≤2500 g). Multiple logistic regression was used to simultaneously control for the confounding effects of all the variables under study and obtain an odds ratio as measure of association. All analyses were carried out with stata, version 7 (18).

The study was designed to detect a two-fold increase in risk with a precision of 0.05 and 80% statistical power. Assuming an estimate of prevalence of LBW (≤2500 g) in Pakistani children at 25% (19) and a 1:1 ratio of depressed and non-depressed mothers, sample size requirements were 154 infants of depressed mothers and a similar number of infants of non-depressed mothers. The Research Ethics Committees of University of Manchester and Rawalpindi Medical College approved the study.

## Results

A total of 701 women in their last trimester of pregnancy (mean 6 weeks from delivery date) were identified. Thirty-one (4%) refused to take part; from the remaining 670, 14 (2%) suffered from a physical disorder and 24 (4%) had other mental disorders (mostly generalized anxiety), and were excluded. Of the 632 women included, 160 (25%) were diagnosed with ICD-10 depressive disorder. Each depressed woman was matched with a psychologically well woman of similar gestation residing in the same Union Council.

At birth, eight infants (3%) (four from the depressed group and four from the non-depressed group) were born prematurely and excluded. Two mothers discontinued the study because of severe depression. Eighteen (6%) (10 from depressed group and eight from non-depressed group) had stillbirths or neonatal deaths, and two newborns (one each from depressed and non-depressed group) had congenital abnormalities and were excluded. Thus 290 mother-infant pairs (143 depressed and 147 non-depressed) were included in the final analysis. One hundred and forty-eight infants (51%) were male and 142 (49%) were female.

Table 1 shows there was no significant difference between the two groups in terms of possession of household assets, type of obstetric care received, sociodemographic factors or maternal height.

**Table 1:** Comparison of depressed and non-depressed groups on household assets, type of obstetric care and sociodemographic characteristics

Household assets, type of obstetric care and maternal factors	Depressed *n* = 143 No. (%)	Not depressed *n* = 147 No. (%)	Chi-square (Mann–Whitney *U* where median)	*P*
Assets				
Electricity	141 (98)	145 (98)	0.000	0.9
A television	51 (36)	66 (45)	2.567	0.1
A bicycle	19 (13)	15 (10)	0.665	0.4
Working on own or family's agricultural land	57 (39)	56 (39)	0.004	0.9
A well with electric pump	35 (24)	36 (24)	0.000	0.9
Toilet with flush	63 (44)	65 (44)	0.000	0.9
Bricks, cement blocks or concrete for walls	133 (93)	142 (96)	1.906	0.1
T-iron, brick or concrete for roofing	121 (84)	130 (88)	0.908	0.3
Delivery care				
Birth attended by a family member	80 (53)	78 (56)	0.242	0.6
Birth attended by traditional birth attendant (TBA)	26 (18)	32 (22)	0.582	0.4
Birth attended by medically trained personnel	37 (25)	37 (25)	0.018	0.8
Maternal factors				
Mothers employed outside home	6 (4)	6 (4)	0.000	0.9
Mothers with no formal education	67 (47)	62 (42)	0.641	0.4
Mothers’ age in years: median [IQR]	26 [24–30]	26 [23–30]	−1.520	0.1
Maternal height in cm: median [IQR]	155 [152–157]	156 [155–157]	1.603	0.1

Infants of depressed mothers were lighter (mean weight 2910 g) compared with non-depressed mothers (mean weight 3022 g). As the data were not normally distributed, Mann–Whitney *U*-test showed that the difference in birthweight between infants of depressed and non-depressed infants was statistically significant (median and interquartile range: 3, [2.6–3.3] vs. 3, [2.5–3.1], *z* = 2.09, *P* < 0.05).

Seventy-five out of 290 infants (25%) in the sample had LBW (≤2500 g). Univariate analysis (relative risk, Fisher's two-sided exact *P*) showed a significant positive association of LBW with antenatal depression and relative poverty, a trend towards a positive association with low maternal BMI and a significant negative association with maternal empowerment (Table 2). None of the other potential risk factors showed an association with LBW.

**Table 2:** Unadjusted relative risk of low birthweight with antenatal depression and other risk factors in 290 newborns

Risk or protective factor	Normal birthweight* (*n* = 215) No. (%)	Low birthweight† (*n* = 75) No. (%)	Relative risk (95% CI)	*P*
Antenatal depression	95 (44)	49 (65)	1.9 (1.3–2.9)	< 0.01
Female gender of infant	106 (49)	36 (48)	0.9 (0.6–1.4)	0.8
Mother's age ≥ 30 years	73 (34)	18 (24)	0.7 (0.4–1.1)	0.1
Mother's age ≤ 20 years	22 (10)	13 (17)	1.5 (0.9–2.4)	0.1
Mother's BMI below 18.5	35 (16)	19 (25)	1.5 (0.9–2.2)	0.08
Mother uneducated	99 (46)	30 (40)	0.8 (0.6–1.2)	0.4
Father uneducated	36 (17)	13 (17)	1.0 (0.6–1.7)	1.0
Being firstborn	30 (14)	14 (19)	1.3 (0.8–2.1)	0.3
Two or more siblings under age 7	129 (60)	45 (60)	1.0 (0.6–1.4)	1.0
Four or more siblings	91 (42)	29 (39)	0.9 (0.6–1.4)	0.7
Living in a nuclear family	68 (32)	17 (23)	0.7 (0.4–1.1)	0.2
Empowerment (mother has some control over finances)	108 (51)	27 (36)	0.6 (0.4–0.9)	< 0.05
At least one antenatal consultation	131 (61)	39 (53)	0.7 (0.5–1.1)	0.2
Relative poverty	70 (33)	37 (49)	1.6 (1.1–2.4)	< 0.01

* More than 2500 g.

† Less than or equal to 2500 g.

Multiple logistic regression (Table 3) showed that after simultaneous adjustment for all risk factors, the association between maternal depression and infant growth remained significant. The only other significant positive association was with relative poverty, while there was no association with any of the other risk factors (data not shown for all factors).

**Table 3:** Estimates of simultaneous effects of antenatal depression and other risk factors in 290 newborns through multiple logistic regression*

	Low birthweight† (*n* = 75)	
	
Risk factor	Odds ratio (95% CI)	*P*
Antenatal depression	2.2 (1.2–4.0)	< 0.01
Relative poverty	2.0 (1.1–3.7)	< 0.05
Being firstborn	2.0 (0.7–5.6)	0.19
Mother's BMI below 18.5	1.6 (0.8–3.5)	0.16
Living in a nuclear family	0.6 (0.3–1.1)	0.12
Maternal empowerment	0.7 (0.4–1.3)	0.31
At least one antenatal consultation	0.7 (0.4–1.4)	0.36

* Data shown for selected factors.

† Less than or equal to 2500 g.

## Discussion

This study shows that maternal depressive disorder in the third trimester of pregnancy is associated with an increased risk of LBW in a low-income developing country, and that this association is independent of the effects of poverty and maternal nutritional status represented by the mother's BMI. In addition, longitudinal follow-up of these infants over a 1-year period showed that maternal depression is associated with growth retardation independent of the effects of LBW (12). Thus, foetal disadvantages associated with maternal depression in pregnancy are two-fold: LBW and subsequent growth retardation. These are accompanied by continued maternal depression during postnatal period (11). These findings must be relevant to the very high but largely unexplained rate of child undernutrition in the region despite adequate food supply.

The study has a number of strengths including a community-based population from a defined geographical rural area of Pakistan, the measurement of maternal depression by experienced clinicians using a standardized instrument and selection of a very similar non-depressed group. The main limitation of the study is that we could not measure haemoglobin or gain any other objective indicator of physical ill health in the mother. It is possible that iron-deficiency anaemia, for example, could be a confounder in the association between depression and LBW. On the other hand anaemia does not appear to be associated with depression (20, 21), and we excluded mothers who said that they had a diagnosed condition or/and regularly took any medication or who had current problems with their pregnancy necessitating medical advice. We also found no significant association between depression or LBW and BMI or maternal height.

Poverty is another potentially important possible confounder. Assessments show only two out of 290 subjects were short of money for food in the previous month indicating hardly any suffered from absolute poverty (22). Our measures of more subtle levels of poverty might be biased as depressed women might selectively recall debt due to their mental state, and may ‘look’ poorer due to their depression. Nevertheless, these were felt to be the best measures because household income can be an unreliable measure in this settings (23). While associations were found between poverty thus measured and LBW, multivariate analyses shows that these do not confound the association seen between maternal depression and LBW in this population. In more impoverished communities, however, poverty may assume a greater role in determining LBW.

Depressed and non-depressed mothers did not differ significantly in their height, precluding it as a confounding factor in this study. Maternal depression was a stronger predictor of LBW than poor maternal nutritional status (defined by a BMI of < 18.5). Poor nutritional status is the principal cause of LBW in developing countries (24) but in the largely food sufficient region of south Asia, maternal depression could play a greater role. Indeed, the reasons for the disproportionately large rates of undernutrition in this region are not fully understood, the problem being referred to as the ‘Asian enigma’ (25). Cigarette smoking and malaria during pregnancy, are other leading causes of LBW (24) but these were not prevalent in our study area. The mean difference of 112 g between babies of depressed and non-depressed mothers gains more significance when the ubiquitous nature and high prevalence of depressive disorder is taken into account. In this study 25% of women were depressed, but in other studies, higher rates have been reported in women with less education and more young children to look after (26, 27). Depression, through its associations with poverty, poor physical health and unhealthy lifestyle (e.g. smoking, poor eating habits, inappropriate health-seeking) could also act as a moderator for these risk factors.

Low birthweight has important long-term health consequences. LWB babies are more likely to have continued growth retardation in early life and poorer intellectual development (28, 29) and are at an increased risk of depression in adolescence (30). Diagnosis and treatment of depression during pregnancy could not only reduce the burden on mothers but could be an important preventive action for both physical and mental health of the off-spring. We are currently involved in a trial of treating depression to see if this improves neonatal outcomes in rural Pakistan.

In developed countries, detection and management of perinatal depression has made significant progress (31). Studies have also shown that greater social support and better psychosocial health facilities for antenatally depressed mothers in low-income communities can lead to improved neonatal outcomes (32, 33). Such psychosocial interventions suitable for developing countries should be developed to tackle these associated problems of immense public health significance. There is the opportunity to take up such an initiative now as part of the goal of reducing LBW incidence by at least one-third between 2000 and 2010, one of the major goals in ‘A World Fit for Children’, the Declaration and Plan of Action adopted by the United Nations General Assembly Special Session on Children in 2002 (1). The reduction of LBW also forms an important contribution to the internationally agreed Millennium Development Goal (MDG) for reducing child mortality and is a key indicator of progress. These goals cannot be achieved by neglecting the mental health of mothers during pregnancy and after childbirth.
